# Research on a Multi-Dimensional Information Fusion Mechanical Wear Fault-Diagnosis Algorithm Based on Data Regeneration

**DOI:** 10.3390/s25123745

**Published:** 2025-06-15

**Authors:** Qifan Zhou, Bosong Chai, Kunwen Ran, Yingqing Guo, Shan Zhou, Wangyu Wu, Kun Wang, Yao Ni

**Affiliations:** 1 School of Power and Energy, Northwestern Polytechnical University, Xi’an 710129, China; george13@mail.nwpu.edu.cn (Q.Z.); 2021302793@mail.nwpu.edu.cn (K.R.); yqguo@nwpu.edu.cn (Y.G.); 2022201871@mail.nwpu.edu.cn (S.Z.); 2 School of Computer Science and Technology, Zhejiang University, Hangzhou 310027, China; chaibosong@mail.zju.edu.cn; 3 School of Computer Science, University of Liverpool, Liverpool L69 3DR, UK; wangyu.wu@liverpool.ac.uk; 4 Institute for Aero Engine, Tsinghua University, Beijing 100084, China; wangk23@mails.tsinghua.edu.cn; 5 School of Integrated Circuits, Guangdong University of Technology, Guangzhou 510006, China

**Keywords:** mechanical wear, diffusion model, test-time training, fault diagnosis algorithms, aero-engine

## Abstract

Under laboratory conditions for recording a small amount of data, the characteristics of the phenomena distribution become a limitation of machine learning and advanced deep learning concepts for the diagnosis and localization of mechanical wear faults. In this paper, we adopt the combination of the diffusion model and TTT (test-time training), based on the sample distribution of feature data under the laboratory conditions, and we use the pre-trained decoder to decode the data into a continuous potential representation of natural language for sampling, to achieve data regeneration. Subsequently, the TTT algorithm becomes a model with weights in the hidden state itself. The gradient step on the self-supervised loss is selected as the update rule, which is trained synchronously during the testing time, adhering to the concept of migration learning, to construct a high-dimensional mapping relationship between the feature parameters and the failure modes of the mechanical wear. The final validation results show that the diagnosis accuracy reaches more than 95% for six types of typical aero-engine mechanical wear faults.

## 1. Introduction

Aero-engines cover a comprehensive framework of thermodynamics, aerodynamics, fluid dynamics, mechanical dynamics, control automation, and mathematical–physical modeling [1]. They are currently one of the most sophisticated pieces of equipment; with the rapid development of technology, aero-engines have also been gradually updated, but also operated in a variety of harsh, demanding, and complex environments [2]. This places higher demands and expectations on the main components, materials, accessories, and health management systems of aero-engines. With aero-engines operating at high temperatures and pressures, at high rotational speeds, and under extreme external influences, the internal mechanical transmission systems are under intense pressure, inflicting wear on and supporting each other, and the lubricants in the lubrication system provide a film of oil that allows for smooth wear on the surfaces of these mechanical transmissions. However, with the prolongation of the operation time, the internal factors of the engine change and the occurrence of fault phenomena such as changes in oil quality, abnormal lubricant leakage, high temperature, and pressure weaken the lubricant volatilization and other factors. With the further aggravation of the wear phenomenon, it evolves into more serious wear failure [3,4,5,6,7,8].

With the rapid iteration and progress of intelligent technology in big data mining and deep learning, many scholars have begun to use these artificial intelligence tools to practically apply mechanical wear algorithms [9]. Algorithm designers have adopted centralized mechanical wear signal processing, characterization under laboratory conditions, particle acquisition by onboard end-of-chip sensors, and the dual acquisition of mechanical wear and signals to achieve mechanical wear fault-pattern recognition and fault-location determination. Through the design of machine algorithms and deep learning frameworks, we can set up a comprehensive idea structure from the acquisition of parameters from the onboard end-of-vehicle chip sensors combined with the fusion of the characteristic parameters from the ground system and the characteristic parameters analyzed in the test-chamber conditions [10,11]. The failure modes are mainly referred to and summarized from the failure modes in the aero-engine mechanical wear failure library, and the typical failure modes are selected and the mapping relationship between them and the failure modes is established by the noising process [12,13,14,15,16,17].

With the rapid development of intelligent manufacturing and Industry 4.0, intelligent health-condition diagnosis techniques for key industrial equipment such as robotic arms and rotary bearings are facing many challenges, including data imbalance, limited labeled data, insufficient cross-field generalization capability, and difficulties in multimodal data fusion. Aiming at these challenges, a series of innovative studies have emerged in recent years: a cross-receptive field-fusion cascade network effectively solves the feature adaptation problem of the robotic arm in migrating a health diagnosis through an adaptive mask-updating mechanism [18]. The study [19] focused on the diagnosis method in highly unbalanced scenarios, respectively, developing a novel intelligent health-state diagnosis framework and a solution based on a graph self-encoder (MNHP-GAE), which significantly improved the recognition rate of a few types of faults. The studies [20,21], on the other hand, focus on fault diagnosis under limited labeled data conditions, with the former effectively alleviating the bottleneck of insufficient supervisory information through a domain knowledge-guided comparative learning framework, and the latter through an adaptive neighborhood-aware comparative network. The study [22,23] further proposes a domain knowledge-driven deep-breadth learning framework, which combines expert experience with deep learning to enhance the model’s interpretability and generalization capability.

In recent years, a large number of scholars and experts at home and abroad have carried out extensive and far-reaching research on mechanical wear fault-diagnosis algorithms for aero-engines and large complex systems [24,25,26]. Experts have used large and complex equipment for the identification of mechanical wear failure modes from a variety of perspectives and technology, such as A.N.Vishwanatha Rao et al. [27]. With an overview and analysis, the study presented the current challenges of gas turbine engines during developmental testing concerning health monitoring instrumentation as a part of health monitoring; special instrumentation systems used to measure various parameters of the rotating components include slip rings, rotational telemetry, and non-intrusive strain measurement systems. The instrumentation of other engine parameters includes values such as temperature, pressure, speed, case vibration, control actuator position, flow rate, clearance between stationary and rotating components, and lubricant quality. Gas turbine engines are complex assemblies of rotating and stationary parts that operate at extreme temperatures, which limits the operational capabilities of the sensors. Error budgets, complete measurement chains, and deriving uncertainties are challenging tasks. Therefore, these scholars have proposed solutions in the areas of engine performance, meta-engine vibration monitoring, lifetime usage monitoring, and rotor support and lubrication system monitoring, respectively [28]; İ Uzun et al. introduced a deep learning-based framework for automatic damage detection from aircraft-engine endoscopy images. The architecture uses a faster R-CNN-based deep learning model with an Inception v2 feature extractor. Due to the limited number of images, data augmentation and other overfitting methods are used. The framework supports crack, burn, notch, and dent damage types for all modules of the turbofan engine. It is trained and validated using moderate to complex endoscopic images obtained from the field [29]; Kumar et al. developed a novel intelligent fault diagnosis and classification method based on a Long Short-Term Memory (LSTM) neural network. The developed model correctly classifies different types of faults under different operating conditions under real operating conditions and the results are compared with the existing techniques. The performance of the model is evaluated using various performance metrics such as precision, recall, F1 score, receiver operating characteristics, area under the curve, and accuracy [30]; Mochamad Denny Surendra et al. used a variety of machine learning algorithms including K Nearest Neighbours (KNN), Support Vector Machines (SVMs), Multilayer Perceptron’s (MLPs) and Decision Trees (DTs) to intelligently monitor abrasive belt-grinding conditions. DT, Random Forest (RF), and XGBoost were used to find machine learning models with optimized hyperparameters that produced the highest average test accuracy. At the same time, DT and RF achieve the lowest latency. Decision tree-based classifiers may be promising models for solving the belt grinding prediction problem.

Li Xueyi et al. came out with a new method called Augmented Depth Sparse Auto Encoder (ADSAE). The method can be used for pitting the fault diagnosis of gears with relatively less raw vibration signal data. The method is mainly based on the theory of pitting fault diagnosis, and creatively combines the idea of data augmentation and depth sparse autoencoder algorithm for gear wear fault diagnosis. The effectiveness of the proposed method is verified through experiments on six gear-pitting conditions. The results show that the ADSAE method can effectively improve the network generalization ability and robustness with very high accuracy [31]. Li Xinglin et al. proposed a novel graph convolutional network (GCN) for HDMS wear fault diagnosis, called Deep Dynamic Higher-order Graph Convolutional Network (DDHGCN). The dynamic graph learning module aims to control the connectivity and sparsity of the iterative graphs to eliminate errors and redundancies caused by noise. A higher-order GCN module is proposed to efficiently model the correlation between nodes, capturing contextual information and their interactions. A residual convolution module is applied to extract local features hidden in a single sample to improve the classification performance further. All three modules are jointly optimized for reliable wear-and-tear fault diagnosis in HDMS.

Measuring wear curves online is often challenging and therefore it is difficult to show the real-time wear state of a bearing. To address this problem [32]. Dai Jingzhou et al. proposed a new digital-twin framework by combining a physically driven model with a data-driven model in parallel. Within this framework, a parallel hybrid model of finite element and deep neural network (PFENN) is built for monitoring and visualizing the real-time wear profile of a plain bearing. In the offline phase, the PFENN obtains the wear distribution by numerical computation of the finite element model and constructs the bearing wear-state space; in the online phase, the PFENN captures the vibration signals of the bearings and obtains the maximum depth of wear by the deep neural network [33]. Guo Yong et al. combined the sound and vibration signals and extracted the eigenvalues required for fusing the signals based on the eigen importance index. The extracted eigenvalues were fused using the concat method to construct sound and vibration fusion vectors, which were combined with a Light Gradient Booster (LightGBM) model for training and diagnosis. A method for diagnosing hydraulic motor plunger wear faults based on the fusion of vibration and sound signal features identified by LightGBM is presented. The method is used to analyze simulated plunger wear test data from an internally curved radial-piston hydraulic motor test rig. The results show that the diagnostic accuracy using the LightGBM algorithm is improved by 1.35%, 2.21%, and 6.92% over the AdaBoost, GBDT, and XGBoost algorithms for the diagnosis of acoustic–vibrational fusion signals affected by the pulsation of the fluid flow [34].

The above scholars in the fields of aircraft engines, large complex equipment and mechanical systems using machine learning algorithms, and deep learning frameworks for the bearings, gears, and end-of-chip characteristics of the parameters of the fault-diagnostic technology research have achieved satisfactory results, most of which focus on the characteristics of the process of mechanical wear and tear of the signal and the data collected by the centralized analysis of the failure modes and characteristics of the comparison. To establish its mapping relationship. The fault-diagnosis logic is divided into two forms based on airborne and ground analysis. Each approach reflects the need for the algorithms to capture, extract, target, and focus on feature information in the diagnostic process. However, there are still some deficiencies in the implementation of these algorithms and the following areas need to be improved:

(1) Data acquisition and analysis methods: the main test of mechanical wear mainly consists of providing the original vibration signal, decomposed into the time–frequency domain feature distribution; obtaining the feature dataset based on iron spectrum analysis, spectral analysis, physical and chemical analysis under the conditions of the test room; and adopting the image detection of mechanical wear and the feature expression of edge distribution. Most of the above-adopted algorithms are only based on vibration signals with a small amount of speech and images for diagnosis. Still, currently, most of the mechanical wear is detected by detecting the on-board end-of-chip sensors and a large number of feature datasets under the laboratory conditions for the reproduction of the failure modes and the high-dimensional mapping, so that the selection of the feature data has become an urgent problem to be solved.

(2) The lack of sample information and weak extraction: because of the process of confidentiality and recording parameters, the number of samples of the analytical feature dataset based on the conditions of the laboratory is small, so it is difficult to extract the in-depth feature expression and semantic information by using a limited number of samples, which has become an uncertainty factor that restricts the accuracy of the final fault-diagnosis results, and it is also the focus of this paper’s research.

(3) Failure mode variability: Under different failure modes, the feature dataset obtained by iron spectrum analysis, spectral analysis, and physical and chemical analysis in the laboratory conditions, there are differences in feature distribution in a large range, but there is a large amount of redundancy in a small range; and in the same kind of failure modes, there are large differences in the feature information; for example, when the severity of the failure modes is different, the features are also large differences. Therefore, an algorithmic model that can learn and update the features continuously is needed to address this disadvantage.

To cope with the deficiencies and drawbacks mentioned above, this paper conducts a demand study on these unresolved problems, to use convergent and innovative algorithms to carry out demand analysis and improvement optimization.

(1) First of all, for the dataset construction problem, this paper adopts the feature dataset based on the fusion of iron spectrum analysis, spectral analysis, and physical and chemical analysis, and takes the real part of the collected dataset as the original database, and adopts the concept of fusion to construct the real dataset first.

(2) In the aspect of data generation, the technique of data regeneration is studied to cope with and solve the relationship between the lack of feature information and the weak semantic expression. The innovative concept of feature data regeneration based on the diffusion model on text features is adopted, with the feature that the continuous diffusion model can be learned in the latent space of the language autoencoder so that the algorithm can sample the continuous latent representations that can be decoded into natural language with a pre-trained decoder, and the bidirectional mode of adding and denoising is utilized to learn and simulate the feature distributions and the trend distributions of the real data to generate the new feature dataset, called the regenerated dataset; the original dataset and the regenerated dataset together constitute a composite dataset.

(3) In solving the problem of complex and variable failure modes, the TTT algorithm is introduced, which adheres to the concept of transfer learning, adopts a test-time training implementation concept, and makes use of the convolutional improvement and substitution in hidden candidates. The algorithm design focuses on solving the fault mode and feature parameters in its fault mode in the same fault feature parameters variable and different fault feature-parameter redundancy of the two limitations of the results of the breakthrough, for the final fault-diagnosis results of the accuracy of the evaluation indexes of the reasonableness and expectations to provide a good solution.

This paper consists of the following elements:

(1) The first part mainly analyses and describes the generation mechanism and evolution cycle of aero-engine mechanical wear failure, and reviews the change process and progress of mechanical wear from the perspective of principle and working condition.

(2) The second part mainly analyses and researches the distribution and trend analysis of characteristic parameters under laboratory conditions based on spectral analysis, iron spectrum analysis, and physical and chemical analysis, and elaborates on the meanings of these characteristic parameters and their relationships with mechanical wear failures.

(3) The third part mainly analyses the key technology research based on the diffusion model and TTT in detail and uses hierarchical algorithm design to elaborate the idea structure and technical solution of mechanical wear fault diagnosis, from the construction of feature dataset to the generation of composite dataset to the final design of the technical route based on TTT algorithm, to provide the basis for the verification of the algorithm.

(4) The fourth part adopts the verification method of evaluation indexes to evaluate the performance of the algorithm in this paper with the indexes of accuracy, recall, F1 score, precision, etc., and conducts comparative analysis with other algorithms to carry out the comparative analysis of the results under the conditions of control variables.

(5) Finally, in the conclusion section, the innovation and advancement of the research content of this paper are elaborated, and the contribution and value of the article are elaborated.

## 2. Mechanical Wear Mechanisms, Evolution, and Analysis of Working Conditions

### 2.1. Analysis of Mechanical Wear Conditions and Working Conditions

The aero-engine is the power source of the aircraft, and its stable and reliable performance is crucial for the flight safety of the aircraft. The mechanical wear of the engine is one of the important factors affecting its performance and reliability [35]. The mechanical wear of the engine occurs for various reasons, mainly including material properties, working environment, operating conditions, design and structure, and other factors [36].

(1). Material characteristics

The engine is composed of complex parts made of various materials, such as blades, turbines, bearings, and so on. Different material characteristics determine its durability and wear performance. The hardness, strength, and fatigue properties of the material will affect its wear under high temperature and high-speed working environment. When the hardness of the material is insufficient or the strength is lower than required for the operating conditions, it will lead to increased mechanical wear of the wearable parts [37,38,39].

(2). Working environment

High temperature, high speed, high pressure, and other factors will cause different degrees of wear and tear on engine components. The softening and thermal expansion of the material in the high-temperature environment will aggravate the wear of the parts; the impact and friction of the gas in the high-speed environment will also accelerate the wear of the parts; the contact area between the parts in the high-pressure environment becomes larger, and the friction increases, which is also prone to lead to the generation of mechanical wear [40,41].

(3). Operating conditions

The engine in different operating conditions will be subject to different workloads, such as starting, accelerating, idling, stopping, and other different stages of the working condition will cause different degrees of wear and tear on the parts. For example, the impact and vibration during start-up will cause minor wear of the parts; the high-speed friction during acceleration will consume the materials on the surface of the parts [42,43,44]; and the continuous work during idling will cause the parts to suffer from wear and fatigue. All of these operating conditions are important causes of mechanical wear.

(4). Design structure

The design structure of components also affects their wear. For example, the surface treatment of the blade, turbine blade shape, bearing lubrication, and other design structures will affect the wear of the components.

For the mechanism of mechanical wear, it is mainly caused by the following three aspects [45,46].

(1). Types of wear

Aero-engine mechanical wear mainly includes abrasive wear, fatigue wear corrosive wear, and other types. Among them, abrasive wear is caused by foreign particles scraping on the surface of the parts; fatigue wear is caused by the expansion of small fatigue cracks produced by the parts in a long period of high-load work; corrosive wear is caused by the medium of chemical corrosion on the surface of the parts.

(2). Wear Kernel

This includes abrasive cutting, wear fatigue, surface fatigue, corrosion, and other major ways. Abrasive cutting is caused by foreign particles in the load and grinding force under the action of scraping the surface of the part; wear fatigue is produced by the parts in the working load of fatigue crack expansion; surface fatigue is produced by tiny cracks in the parts’ surface of the working load expansion; corrosion is caused by the corrosion of chemical media on the surface of the parts [47].

(3). Wear control

Mechanical wear in aviation engines can be from the material selection, surface treatment, design structure, lubrication system, and other aspects of wear control. The selection of high hardness, high strength, and high wear-resistant materials can reduce the wear rate; surface coating, surface shot blasting [48,49,50], and other treatment methods can improve the surface quality of parts; the design of a reasonable structure can reduce the degree of wear; optimizing the lubrication system can reduce the friction of the contact surface of parts.

### 2.2. Evolution of Mechanical Wear Under Changing Operating Conditions

A long period of operation is subject to a variety of operating conditions that lead to the development of mechanical wear. The evolution of mechanical wear is a complex kinetic process that is influenced by a variety of factors.

(1). The evolution of mechanical wear under high-temperature and high-pressure conditions

In the operation of an aero-engine, high temperature and high-pressure working conditions are the most common working conditions. Under high temperatures and high pressure, the thermal and mechanical stresses inside the aero-engine will increase, which leads to the increased wear of mechanical parts. Under this condition, the evolution of mechanical wear can be divided into the following stages:

Surface initial wear: Under high-temperature and high-pressure conditions, the surface of mechanical components will be subject to strong impact and friction, resulting in surface initial wear. This kind of wear is mainly caused by the interaction and relative motion between particles, which will lead to particle wear and micro-cracks on the surface of engine parts.

The generation and accumulation of wear particles: in high-temperature and high-pressure conditions, mechanical parts of the wear particles will gradually be generated and accumulated on the surface. These wear particles will further aggravate the wear of mechanical parts, forming a vicious circle [51].

The formation of a surface wear layer: With the continuous generation and accumulation of wear particles, the surface of mechanical parts will gradually form a wear layer. This wear layer will accelerate the wear of mechanical parts, resulting in increased roughness of the surface of the parts, further aggravating the wear.

The shedding of wear particles and material expansion: In high-temperature and high-pressure working conditions, the surface wear layer of mechanical parts will gradually fall off, and wear particles will also fall off. At the same time, the thermal expansion of the material will accelerate the surface wear of mechanical parts, leading to a decline in the relevant performance of mechanical parts.

Failure of mechanical parts: Under high-temperature and high-pressure working conditions, the increased wear of mechanical parts, increased surface roughness, material shedding and thermal expansion, and other factors will lead to the failure of mechanical parts, thus affecting the normal operation of the aero-engine [52].

(2). The evolution of mechanical wear under low-temperature and low-pressure working conditions in the operation of aircraft engines, low-temperature and low-pressure working conditions are also common working conditions. In this condition, although the mechanical parts are subjected to thermal stress and mechanical stress is small, the evolution of the mechanical wear process still has certain characteristics [53,54].

Surface adhesion wear: in low temperature and low-pressure working conditions, the surface of mechanical parts will appear adhesion wear, mainly due to surface roughness, surface hardness, load and temperature, and other factors. This kind of wear will lead to surface abrasion and microscopic deformation of mechanical parts, thus affecting their relevant performance.

Surface fatigue wear: under low-temperature and low-pressure working conditions, the surface of mechanical parts will be subject to fatigue loading, resulting in the formation of fatigue wear. This kind of wear is mainly due to the gradual expansion of microscopic cracks on the surface, resulting in brittle damage to the surface materials of mechanical parts, thus causing wear.

Generation and accumulation of wear particles: In low-temperature and low-pressure working conditions, the generation and accumulation of wear particles in mechanical components is also inevitable. These wear particles will further aggravate the wear of mechanical parts, thus affecting their service life and reliability.

Surface wear-layer formation: with the continuous generation and accumulation of wear particles, the surface of mechanical parts will gradually form a wear layer. This wear layer will accelerate the wear of mechanical parts, increasing the roughness of the surface of the parts, and further aggravating the wear [55,56,57].

The failure of mechanical parts: under low temperature and low-pressure working conditions, the wear layer of mechanical parts gradually increases, wear particles continue to accumulate, and surface adhesion wear and fatigue wear under the joint effect will lead to the failure of mechanical parts, affecting the normal operation of the aero-engine.

(3). The evolution of mechanical wear under other special working conditions in addition to high-temperature and high-pressure working conditions and low-temperature and low-pressure working conditions: the aero-engine will also be affected by other special working conditions, resulting in the evolution of mechanical wear. For example, mechanical wear under wet environments, and mechanical wear under high-speed movement.

In a wet environment, mechanical components are lubricated by a water film, which affects the evolution of mechanical wear. Under these conditions, the surfaces of mechanical components are subjected to impact and friction by the water film, leading to surface wear and corrosive wear. This wear leads to an increase in the surface roughness of the parts, thus affecting their reliability and service life.

### 2.3. Overview of the Mechanical Wear Dataset

Mechanical wear failure modes are diagnosed under laboratory conditions using feature datasets based on iron spectral analysis, spectral analysis, and physicochemical analysis to jointly diagnose the final possible failure modes. Six features, namely, normal sliding, severe sliding, cutting abrasions, fatigue abrasions, laminar abrasions, and globular abrasions, are used as part of the feature dataset for iron spectral analysis, while six features, namely, Fe, Ag, and Cu, are used as part of the feature dataset for spectral analysis. Cu, Cr, Mo, V, O, Zn, Sn, and other metal elements were selected as part of the feature dataset for spectral analysis, while viscosity, acidity, and flash point were used as part of the feature dataset for physicochemical analysis; the last part of the feature dataset was based on the collection of features from the on-board end-of-chip sensors, and the particulate analysis was used as a feature to characterize the particulate matter sizes of 5 µm, 15 µm, 25 µm and 50 µm. as feature datasets [58].

**Figure 1:**
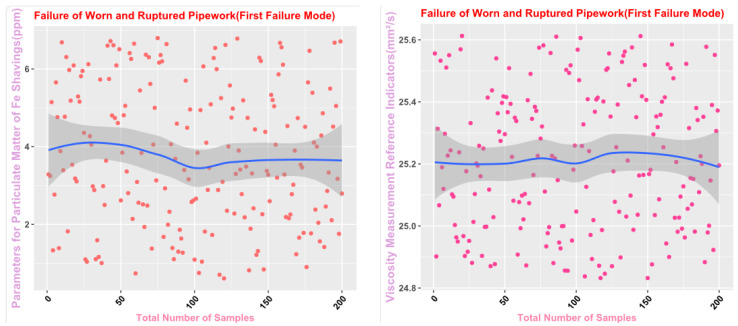
The distribution of feature parameters in the fault of worn and ruptured pipework model.

**Figure 2:**
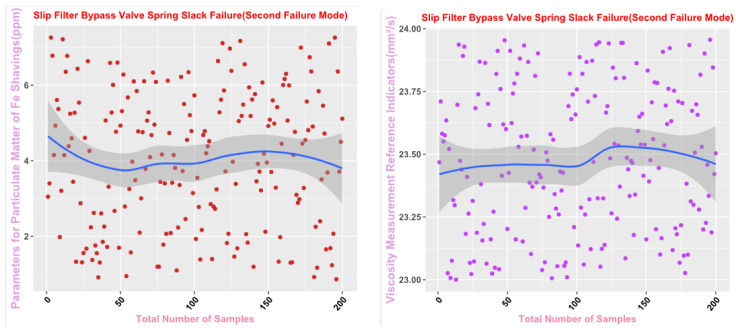
The distribution of feature parameters in the slip filter bypass-valve spring slack fault.

**Figure 3:**
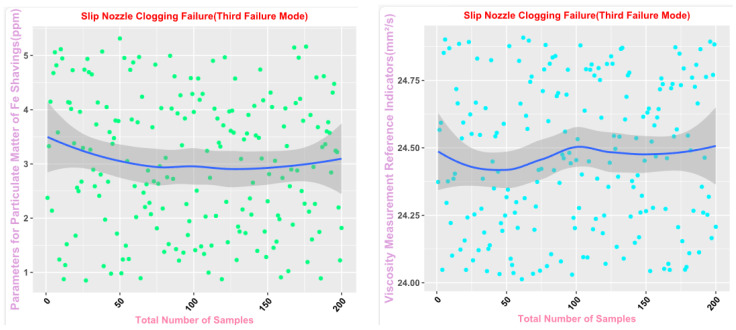
The distribution of feature parameters in the slip nozzle clogging fault.

**Figure 4:**
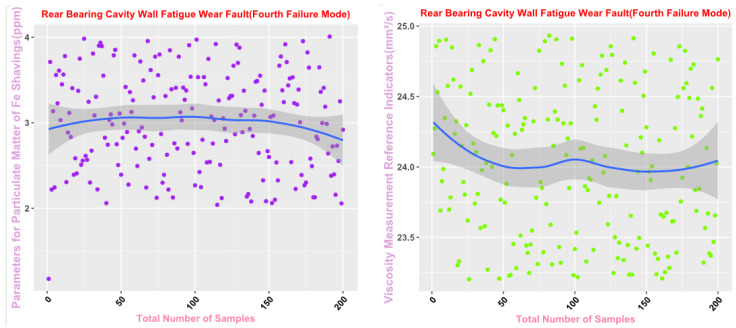
The distribution of feature parameters in the rear bearing cavity-wall fatigue wear fault.

**Figure 5:**
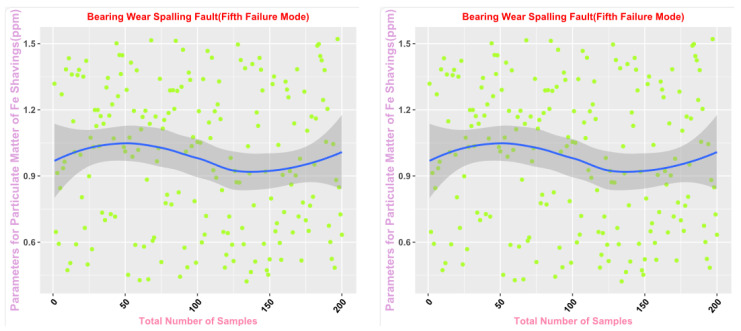
The distribution of feature parameters in the bearing wear spalling fault.

**Figure 6:**
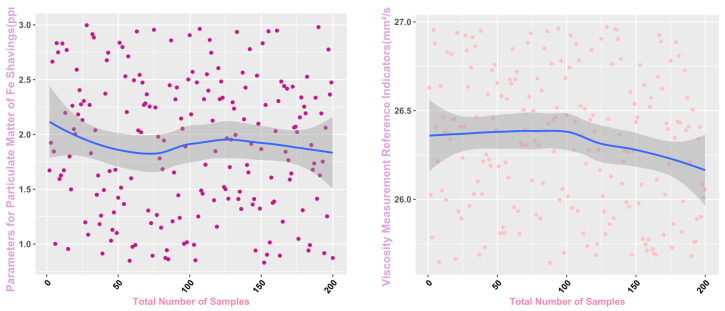
The distribution of feature parameters in the gearbox-gear heavy wear fault.

Some of the data from the Mechanical Wear Characteristics dataset is shown in Table 1, Table 2, Table 3 and Table 4, and the trend distribution and discrete feature distribution are shown in Figure 1, Figure 2, Figure 3, Figure 4, Figure 5 and Figure 6.

Under laboratory conditions, the mechanical wear fault-diagnosis dataset constructed in this study is derived from the results of accelerated aging tests at the University of Civil Aviation and in the aerospace field. By simulating the physical processes of six typical failure modes (e.g., the heavy wear of gears in gearboxes, the fatigue wear of bearing cavity walls, etc.), the 18-dimensional spectral feature data are systematically collected and analysed using the following equipment and methods: (1) Vibration signal acquisition: using a Kistler 8728A triaxial acceleration sensor (frequency response range 0.5 Hz∼10 kHz%) and NI cDAQ-9188 data acquisition system (sampling rate 51.2 kHz), installed in the gearbox bearing housing and shell key measurement points, combined with the ISO 10816 standard vibration intensity analysis, through the wavelet packet transform (WPT) to extract the Normal Slide, Severe Slide, Slide and Severe Slide data. Slide, Severe Slide and other time–frequency features are extracted by Wavelet Packet Transform (WPT), and Cutting Grain impact components are identified based on Kurtosis and Envelope Demodulation; (2) physical and chemical characteristics of the oil monitoring were determined by using an On-line VISCOPro 2000 Viscometer (accuracy 1%), a Metrohm 902 pH meter (resolution 0.01), and MINIFLASH FLP fully automated monitoring. MINIFLASH FLP full-automatic flash-point meter (ASTM D7094 standard), the real-time recording of lubricating oil viscosity, acidity, and flash point of the continuous changes in the trend, and at the same time, through the OLI lubricating oil spectral analyser (wavelength range of 190∼900 nm) every two hours to collect Fe, Ag, Cu and other metal element concentration (ppm) Meanwhile, the concentration of Fe, Ag, Cu and other metal elements (ppm) is collected every 2 h by OLI lubricant spectral analyzer (wavelength range 190∼900 nm), and the size distribution and morphology of wear particles (e.g., Spherical Grits, Laminated Grit) are evaluated by combining with the ISO 4406 standard rated load, and adopting fault implantation technology—including controlled abrasive injection (FZG standard test dust), spring preload adjustment (slip filter bypass-valve failure), nozzle clogging simulation (orifice step reduction), and an accelerated fatigue test (high-frequency alternating stress loading)—to synchronously collect multi-source data to build a test rig covering the entire life cycle. The data is collected simultaneously from multiple sources to build a sample library of 3200 groups covering the entire life cycle.

There are multiple levels of correlation between the six mechanical failure modes and the characterization data, with different faults triggering specific wear characteristics, elemental changes, or lubricant degradation to form diagnosable failure modes. The fault of the worn and ruptured pipework model is often accompanied by an abnormally high Fe and Cu elemental content due to metal pipework wear and a significant drop in viscosity (lubricant contamination), while the slip nozzle clogging fault is significantly associated with Cutting Grain and O, Sn elements (oxidation deposits). Viscosity decreases (lubricant contamination), while the slip nozzle clogging fault is significantly associated with Cutting Grain and O and Sn elements (oxidized deposits), while elevated acidity accelerates the clogging process. Wear fault and bearing wear spalling fault are both characterized by an increase in Fatigue Grit and Spherical Grits (fatigue spalling particles), but the latter is also characterized by an abnormal concentration of the corresponding elements due to spalling of bearing alloys such as Cr and Mo. Gearbox-gear heavy wear fault is characterized by Severe Sliding Grit, and O fault is characterized by the buildup of Laminated Grit under Severe Slide conditions, accompanied by changes in the concentration of V and Zn elements (gear additive wear). Additionally, the indirect correlation of the slip filter bypass-valve spring slack fault is characterized by a decrease in flash point (lubricant degradation) and abnormalities in Ag elements (valve body plating wear). These correlations indicate that the failure modes do not exist in isolation from the characterization data, but are dynamically mapped through the coupling of wear mechanisms (e.g., abrasive morphology), material loss (elemental composition), and lubrication properties (viscosity, acid value), providing an interpretable physical basis for multimodal data-based fault diagnosis.

## 3. A Technical Route for Implementing Mechanical Wear Fault Diagnosis Based on the Fusion of the Diffusion Model and TTT

### 3.1. Research on Key Technology of Diffusion Model Based on Data Regeneration

The diffusion model, as an implicit variable model, focuses on learning the random Gaussian noise in the feature parameters, and iteratively converts the random Gaussian noise (using analytical sampling) from wear feature sample parameters to samples from an unknown data distribution specified in the sample set. This high-dimensional mapping is defined using an iterative process of forward diffusion to add Gaussian noise to the set of feature-parameter samples, and an iterative process of generating new samples from denoised feature-parameter samples from the Gaussian distribution. The diffusion model consists of a denoising network ${\hat{x}}_{\theta }$ with the following network structure [59,60,61]:

(1) $$L\left(\theta \right)=E_{t,x,\delta }\left[\lambda_{t}{∥{\hat{x}}_{\theta }\left(\sqrt{\alpha_{t}}x+\sqrt{1-\alpha_{t}}\delta ,t\right)-x∥}_{2}^{2}\right]$$

where X is the sample set of wear feature parameters used for training, t∼U(0,1) is the time-step metric, δ∼N(0,1) is the Gaussian white noise distribution, $\alpha_{t}$ defines the white noise schedule, and $\lambda_{t}$ is the time-dependent weighting term. Because of this, the denoising process is trained to denoise the noisy hidden signal $z_{t}=\sqrt{\alpha_{t}}x+\sqrt{1-\alpha_{t}}\delta$ to a trace noisy feature sample X. The accompanying regression target emphasizes a specific time *t*. The target sampling starts with pure Gaussian noise $z_{1}∼N\left(0,1\right)$, and the denoising network is utilized to iteratively generate the latent value $z_{1},z_{2},\ldots ,z_{T}$, where the $z_{0}$ noise level is progressively reduced until it is approximated from the data distribution [62,63].

**Figure 7:**
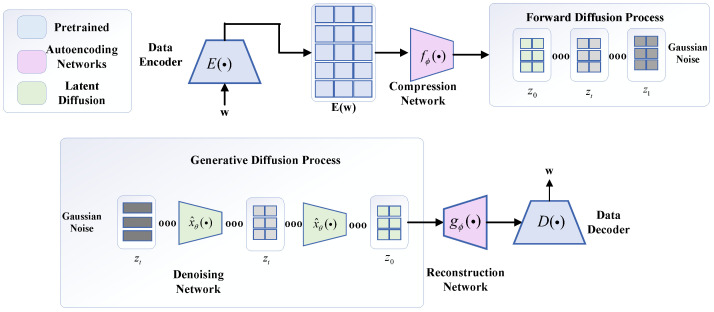
An analysis and investigation of the principles.

The one-dimensional feature data-generation model used for data generation is shown in Figure 7, which significantly expresses the potential diffusion process of one-dimensional detected data, and consists of two main components and the implementation of key techniques. The codec language model used for *pre-training* is augmented using two network models capable of performing continuous learning tasks. Afterward, a continuous diffusion model is used to construct composite features richer in semantic information by exploiting the information distribution of existing real feature datasets and thus generating new samples.

The data-generation diffusion learning model is mainly carried out with pre-trained coder–decoder connections, where the pre-training process is brought to a standstill in the regular mode, and learning is carried out using the auto-coding module.

Inside the framework, the core architecture consists of a compression network model, which maps the corresponding mechanical wear features and internal latent information of the encoder model into a diffuse and compact latent space. The learnable compression network model adopts a perceptron resampling model structure, which is shown in Figure 7, and its structure consists of a multi-head attention mechanism and a feed-forward network together, which is continuously traversed by using the querying of the hidden state, and iteratively repeated cross-learning, to identify the core attention target in the data encoder of the mechanical wear features, to extract the key useful potential information. In the meantime, consistent to that elaborated above, the potential queries are focused on their own useful and important feature information and stagnate the semantic representation of the encoder, where the expression for the attention mechanism is shown in Equation (2).

(2) Z=Z+MHA(q=Z,kv=[Z;E(w)])

where MHA(·) has mechanisms for query *q* and key/value *attentional* representations. The encoder is represented as compressed to a potentially fixed sequence length, and a *feedforward* layer is applied to the potential query representation after each multi-head attention layer.

**Figure 8:**
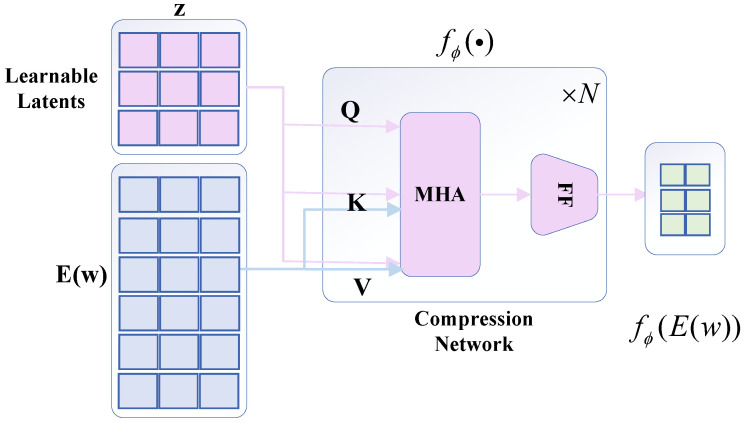
Schematic diagram of compression network structure.

As shown in Figure 8, after the compression network maps the input sequence of mechanical wear feature information inputs to a set fixed length sequence, the linear projection of the incidental learning mechanism is used to reduce the dimensionality of the output information to a set dimensionality, at which point the compression network model maps the stagnant encoder variable sequence outputs to a compact potential space.

(3) $$x=f_{\phi }\left(E\left(w\right)\right)\in R^{d_{z}\times d_{x}}$$

Next, we will cover the utilization of the diffusion model, where the potential space needs to be *synergistically* scaled accordingly to achieve diffusion, hence the need to limit and set the number of paradigms of the potential space to ensure this realization of the concept. When $E_{\delta ∼N(0,I)}{[∥\delta ∥}_{2}^{2}]=d_{ae}$ is at the level of feature dimensional consistency, the intrinsic feature vectors are normalized for diffusion.

The main key architecture of the post-compression network execution process mainly utilizes the reconfiguration network model, which, as the name suggests, mainly maps the potential space of the compressed network model in the direction of the data decoder’s needs and set expectations, utilizes $x=f_{\phi }\left(E\left(w\right)\right)\in R^{\ell \times d_{3c}}$ for the back-projection to dimension $d_{LM}$, and adds an embedded location process with a dependency learning mechanism to it, and utilizes the Transformer model to obtain $g_{\phi }\left(x\right)\in R^{\ell \times {\hat{d}}_{LM}}$. D(·) in the data decoder cross-processes these mechanical wear features and information features, and autoregressively generates the corresponding one-dimensional data form. Using training compression, the reconstruction network is used to generate these similar features and directs the decoder to reconstruct the input feature information with cross-entropy loss $w\approx D\left(g_{\phi }\left(x\right)\right)=D\left(g_{\phi }\left(f_{\phi }\left(E\left(w\right)\right)\right)\right)$.

The ultimate aim and need of the diffusion model for data generation is to regenerate new data, thereby increasing the intrinsic availability of data at the level of semantic and feature information to build composite datasets. In the *denoising* network structure model *A*, absolute positional coding with dependency learning with the *GeGLU* activation function is utilized. This activation is utilized to extend and improve the diffusion performance and applicability. The adaptive *autoencoder* hidden unit is mapped to the Transformer dimension and then, after the co-processing process of the Transformer, it is reflected and projected back to the adaptive *autoencoder* hidden unit dimension, thus obtaining the final result. Subsequently, α*-tuning* is utilized to modify and adapt to the noise variations. The $\alpha_{t}$ is mapped to a sinusoidal positional *embedding* and passed through an MLP with a single hidden layer to obtain a temporal embedding. This temporal embedding is added to the input sequence and adaptive-layer normalization conditional on the temporal embedding is applied to the output of each *feedforward* layer.

In summary, the diffusion model is a latent variable model architecture with latent variable $z=\{z_{t}|t\in \left[0,1\right]\}$, q(z∣x) where data x∼p(x) comes from the distribution of mechanical wear feature information of the location, and the forward process follows a Markov process, where Gaussian noise is *iteratively* and continuously added to the raw data present as the algorithm’s execution process progresses.

(4) $$q\left(z_{t}|x\right)=N\;\left(z_{t};\sqrt{\alpha_{t}}\;x,\left(1-\alpha_{t}\right)\;I\right),\;q\left(z_{t}|z_{s}\right)=N\;\left(z_{t};\sqrt{\alpha_{t|s}}\;z_{s},\left(1-\alpha_{t|s}\right)\;I\right)$$

where $\alpha_{t|s}=\alpha_{t}/\alpha_{s}$ and 0≤s<t≤1, and the noise variation of $\alpha_{t}\in \left[0,1\right]$ becomes smaller with t, and the final potential time resembles a Gaussian distribution, and $q\left(z_{1}\right)\approx N\left(z_{1};0,I\right)$ is independent of the original mechanical wear feature data, then the forward process defines the process by which the original feature data distribution changes to a Gaussian distribution. For the potential linkage of the original mechanical wear characterization data, the corresponding reverse study is carried out using the parsing of the forward process, and for t>s, there exists $q\left(z_{s}|z_{t},x\right)=N\left(\mu_{Q}\left(z_{t},x,s,t\right),\sigma_{Q}^{2}\left(s,t\right)I\right)$, $\mu_{Q}\left(z_{t},x,s,t\right)=\frac{\sqrt{\alpha_{s}\left(1-\alpha_{t|s}\right)}}{1-\alpha_{t}}\;x+\frac{\sqrt{\alpha_{t|s}\left(1-\alpha_{s}\right)}}{1-\alpha_{t}}\;z_{t}$, $\sigma_{Q}^{2}\left(s,t\right)=\frac{\left(1-\alpha_{s}\right)\left(1-\alpha_{t|s}\right)}{1-\alpha_{t}}$, which is the reverse data-regeneration process in the diffusion model, which is unavailable in the generation process. Machine learning is used to infinitely approximate the potential distribution of noise given the original mechanical wear feature data at the time step ${\hat{x}}_{\theta }\left(z_{t},t\right)\approx x$, while the denoising network is trained using the regression loss principle for the training process:

(5) $$L\left(\theta \right)=E_{t,x,\hat{o}}\;\left[\lambda_{t}\;{∥{\hat{x}}_{\theta }\left(\sqrt{\alpha_{t}}\;x+\sqrt{1-\alpha_{t}}\;\hat{o},\;t\right)-x∥}_{2}^{2}\right],$$

This loss function has certain time-dependent weights *A* and can be motivated as a weighted variational lower bound on the log-likelihood of the data under a forward diffusion process. The *denoising* network is generally parameterized as a prediction network, where the velocity v is defined as $v=\sqrt{\alpha_{t}}\hat{o}-\sqrt{1-\alpha_{t}}\;x$. These parameterizations can be interpreted as different weighting functions $\lambda_{t}$ for the regression objective. The principle of this *parameterization* is used in the diffusion model’s implementation process, whereby the *denoising* network is trained, and the whole data-regeneration process is then defined:

(6) $$p_{\theta }\left(z_{s}|z_{t}\right)=N\left(z_{s};\mu_{\theta }\left(z_{t},s,t\right),\sigma^{2}\left(s,t\right)I\right)$$

where

(7) $$\mu_{\theta }\left(z_{t},s,t\right)=\mu_{Q}\left(z_{t},{\hat{x}}_{\theta }\left(z_{t},t\right),s,t\right),\;\sigma^{2}\left(s,t\right)=1-\alpha_{t|s}$$

Ultimately, the variance of $p_{\theta }\left(z_{s}|z_{t}\right)$ is set to be $\sigma^{2}\left(s,t\right)=1-\alpha_{t|s}$ for the original level of data estimation brought in the posterior distribution $q(z_{s}|z_{t},x)$, thus parameterizing the mean $p_{\theta }\left(z_{s}|z_{t}\right)$ of the generation process of the brand new feature data, following and depending on the selection process of the variance parameter in the forward process. In the data-regeneration process, the initial noise $z_{t_{1}}=z_{1}∼N\left(0,I\right)$ is sampled using the DDPM sampler, and the update selection is carried out iteratively using polling:

(8) $$z_{t_{i+1}}=\mu_{\theta }\left(z_{t_{i}},t_{i+1},t_{i}\right)+\sigma \left(t_{i+1},t_{i}\right)\delta$$

δ∼N(0,I) and the interrupt time step $1=t_{1}>t_{2}>\ldots >t_{T}=0$ are interpolated between 0 and 1 for calculation [64].

### 3.2. Key Technology Research on Test-Time-Training-Based Fault-Diagnosis Algorithm Model

The TTT algorithm combines the excellent performance of the self-attention mechanism in the context of data features with the linear complexity that the RNN algorithm layer has. On the one hand, the broad sensory field acquisition of the context and the multidimensional feature weighting for the need to focus on the high-dimensional mapping logic between the mechanical wear feature information and the failure modes is first established, and secondly, the advantage of RNN in linear complexity performs the extraction of the mechanical wear feature signal information, the deep feature mapping mining and the correspondence between the failure modes further clarified. The TTT algorithm is an organic combination of these great advantages: firstly, the TTT algorithm has the linear complexity of the new serialization modeling layer and the expressive hidden state; secondly, the hidden state itself becomes a machine learning model, updating rules as a part of self-supervised learning, so in the process of the test, it also acquires the ability to train to the hidden candidate state. The TTT is therefore adaptable to identify and localize fault patterns for different mapping relationships [64].

**Figure 9:**
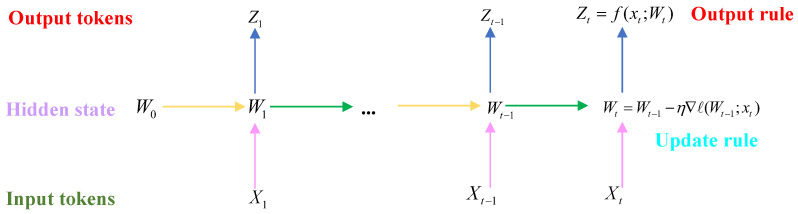
TTT algorithm architecture and its update rule flow.

The structure of a typical TTT algorithm is shown in Figure 9, where the serialized model architecture represents a layer that can transform the hidden candidate states according to the update rules. The TTT algorithm makes the hidden candidate states themselves into algorithmic models with weights W, and the prior criterion for updating is the gradient step size in a self-supervised learning task. This architectural concept of iteratively updating the hidden candidate states in the test task is equivalent to training the algorithmic model while testing the mechanical wear feature data, thus building the TTT layer structure [65].

The process of learning the wear characteristics or updating and refining the parameters within the algorithm is to centrally store and compress a large set of training model characteristics into the weights of the algorithmic model so that a large number of parameters can be used to establish a mapping to the failure modes to adapt to small or large changes. Therefore, using self-supervised learning, the multi-feature semantic and contextual feature information $x_{1},\ldots ,x_{t}$ is centrally compressed and stored, the hidden candidate state $s_{t}$ is equivalently represented as $W_{t}$, and the algorithmic model weights are represented as a small neural network model. The output rule of the algorithmic model follows the following equation:

(9) $$z_{t}=f\left(x_{t};W_{t}\right)$$

The output will be the algorithmic model making predictions about the input set of mechanical wear feature parameters $x_{t}$ using continuously updated iterative weights $W_{t}$, while the update rule processes the gradient descent of the self-supervised loss function.

(10) $$W_{t}=W_{t-1}-\eta \nabla \ell \left(W_{t-1}\;\cdot \;x_{t}\right)$$

In terms of the compressed and centralized processing of mechanical wear feature data, the algorithm’s memory population is capable of autonomously deciding whether to remember or forget the feature distribution of the input and $x_{t}$ is capable of storing and remembering inputs with large gradients. To achieve this, input B needs to be reconstructed and the input set to a bad situation.

(11) $$\ell \left(W;x_{t}\right)={∥f\left({\bar{x}}_{t};W\right)-x_{t}∥}^{2}$$

The self-attention mechanism of the TTT algorithm is more similar to that of the RNN, where the algorithm maps the mechanically worn input feature dataset $x_{1},\ldots ,x_{T}$, and compiles the output sequence features $z_{1},\ldots ,z_{T}$ using the corresponding hidden candidate states, more refined rules, and output logic into the forward pass process of the algorithmic model, so that when the algorithmic implementation is carried out on the test set, the new layer is still able to be trained on the different weights $W_{1},\ldots ,W_{T}$, which is then defined as the TTT layer.

The most central task of the TTT algorithm is self-supervised learning, where an end-to-end optimized self-supervision approach is taken to be able to obtain richer and more realistic prior knowledge. The raw feature information input of mechanical wear is turned into a low-rank projection ${\bar{x}}_{t}=\theta_{k}x_{t}$, $\theta_{k}$ is the matrix available for self-learning, $\theta_{k}x_{t}$ is named as the training attempt, and the key concerns in the feature information of mechanical wear are not all memorized. Still, they are targeted to be selectively extracted and constructed into the mapping relations. Thus, the labels are reconstructed as another low-rank projection $\theta_{v}x_{t}$. The final loss for the self-supervised learning task is as follows:

(12) $$\ell \left(W;x_{t}\right)=f\left(\theta_{k}x_{t};W\right)-\theta_{v}x_{t}^{2}$$

In the output rule, weight *W* is optimized and thus is an internal parameter of *ℓ*. Whereas $\theta_{s}$ is a hyperparameter in the overall algorithm, in the external iteration process of the whole algorithm model, $\theta_{k},\theta_{v},\theta_{o}$, and $\theta_{\mathrm{rest}}$ are all iteratively optimized, at which point weight *W* becomes a candidate hidden state. So the new output rule is then:

(13) $$z_{t}=f\left(\theta_{Q}x_{t};W_{t}\right)$$

The training and failure mode labeling views of the feature dataset compress into $W_{t}$ and propagate forward in time the information in the mechanical wear feature parameter $x_{t}$. The test view of the feature dataset maps to the current output *C* and propagates forward through the network layer with potentially different information. The test view of the feature dataset maps to potentially different information in the current output $z_{t}$ and propagates forward through the network layer. The set of all possible choices of $\theta_{K},\theta_{v},\theta_{Q}$ induces a family of multi-view reconstruction tasks, where all views are designed as linear projections.

Whereas the TTT algorithm is more innovative and advantageous in terms of time complexity and expression of floating-point operations and speed, the implementation of this algorithm adheres to the concept of parallelization, where gradient descent provides the basis for this improvement, where the update rule for gradient descent is expressed as:

(14) $$W_{t}=W_{t-1}-\eta G_{t}=W_{0}-\eta \sum_{s=1}^{t}G_{s}$$

**Figure 10:**
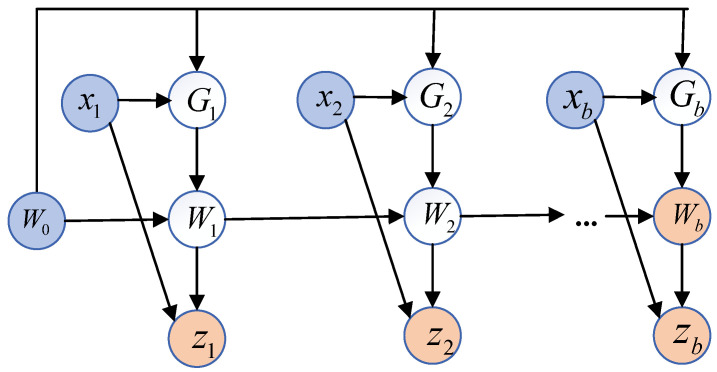
Mini-TTT batch higher-order computing architecture.

Within the TTT algorithm, the optimization concept of the small-batch gradient descent method is used as shown in the figure below, where *b* denotes the number of quantities used to batch the TTT algorithm, utilizing $G_{t}=\nabla \ell \left(W_{t};x_{t}\right)$. $t^{\prime }=t-\mathrm{mod}\left(t,b\right)$ is the small-batch processing for the previous time step, and for the latter time step, parallel processing is performed utilizing multiple gradient computations. The different variants of gradient descent affect only the gradient channel, i.e., the descent direction $G_{t}$ and, in particular, w.r.t., where *W* is the gradient. However, the descent step $W_{t}=W_{t-1}-\eta G_{t}$ always starts from $W_{t-1}$ due to the autoregressive nature of the update rule, which is orthogonal to the choice of $G_{t}$. Figure 10 illustrates the mini-TTT batch higher-order computing architecture.

The most important advantage of the TTT algorithm is the design of its dyadic form, which provides an ideal implementation of the wall clock and a breakthrough in the efficiency of the algorithm execution. The loss at time *t* is:

(15) $$\ell \left(W_{0};x_{t}\right)={∥f\left(x_{t};W_{0}\right)-x_{t}∥}^{2}={∥W_{0}x_{t}-x_{t}∥}^{2}$$

The expression after parallel computation is shown in the following equation:

(16) $$G_{t}=\nabla \ell \left(W_{0};x_{t}\right)=2\left(W_{0}x_{t}-x_{t}\right)x_{t}^{T}$$

In order to reduce computational consumption and speed up the execution of the algorithm, TTT uses unspecified $G_{1},\ldots ,G_{b}$, computes the weights $W_{b}$ at the same time when the small batch is over, and serializes the output $z_{1},\ldots ,z_{b}$.

(17) $$W_{b}=W_{0}-\eta \sum_{t=1}^{b}G_{t}=W_{0}-2\eta \sum_{t=1}^{b}\left(W_{0}x_{t}-x_{t}\right)x_{t}^{T}=W_{0}-2\eta \left(W_{0}X-X\right)X^{T}$$

At this point, $W_{b}$ is able to iteratively update and compute $Z=[z_{1},\ldots ,z_{b}]$.

(18) $$z_{t}=f\left(x_{t};W_{t}\right)=W_{t}x_{t}=\left(W_{0}-\eta \sum_{s=1}^{t}G_{t}\right)x_{t}=W_{0}x_{t}-2\eta \sum_{s=1}^{t}\left(W_{0}x_{s}-x_{s}\right)x_{s}^{T}x_{t}$$

And, by passing $\delta_{t}=\sum_{s=1}^{t}\left(W_{0}x_{s}-x_{s}\right)x_{s}^{T}x_{t}$ and $\Delta =[\delta_{1},\ldots ,\delta_{b}]$, the result is obtained as shown below:

(19) $$\Delta =\left(W_{0}X-X\right)\mathrm{mask}\left(X^{T}X\right)$$

where the mask identifier is an upper triangular mask with zeros, and $W_{0}X-X$ can be reused in the calculation of $W_{b}$. Using the calculation of mammals, $Z=W_{0}X-2\eta \Delta$ is calculated by substituting η into Equation (18). The computation proceeds in dyadic form, where $G_{s}$ and $W_{s}$ are explicitly materialized in the original form. These two forms are equivalent in output [66,67,68].

### 3.3. Implementation Architecture of Mechanical Wear Fault-Diagnosis Algorithm Based on the Fusion of Data Regeneration and TTT Algorithm

The architecture of the mechanical wear fault-diagnosis algorithm based on the fusion algorithm is shown below, in which the algorithm implementation structure mainly includes two parts of the core technology implementation. The first part involves the use of the diffusion learning algorithm to regenerate the fusion dataset information based on the four key features of iron spectral analysis, spectral analysis, physical and chemical analysis, and particle diameter under the laboratory conditions, to construct a composite dataset that fuses the original mechanical wear data with the regenerated data; the second part involves the use of the composite dataset as an input for the execution of the TTT algorithm, and to take advantage of the key innovations and advantages of TTT algorithm in terms of time complexity, space complexity, and test-time training. In the second part, the composite dataset is used as the input for the TTT algorithm, which makes use of the key innovations and advantages of the TTT algorithm in terms of time complexity, space complexity, and training at test time, and adheres to the concept of transfer learning to accurately identify and locate the results of the failure modes and centrally display the results and verify them with the corresponding evaluation indicators and comparative analysis.

**Figure 11:**
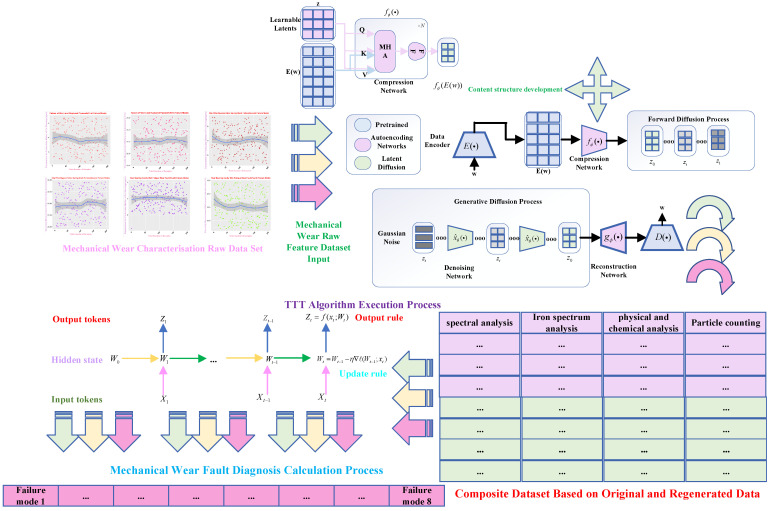
The flow of the implementation architecture of the mechanical wear fault-diagnosis algorithm based on the fusion of the diffusion model and TTT algorithm.

Figure 11 analyses and investigates the principles of fault-diagnosis algorithm implementation according to a hierarchical execution architecture process, following the steps and relevant details, hyperparameter selection, data integration analysis, and algorithm fusion concepts.

The algorithm execution process in the core execution process is mainly divided into two parts; the first is the original dataset construction through the diffusion model for data regeneration to combine into a composite dataset process, the original dataset part includes an iron spectral analysis of abrasive characteristics of the information, a spectral analysis of the characteristics of the metal element information, a physical and chemical analysis of the viscosity, acidity, and flashing characteristics of the information, and the particle counts include a variety of abrasive particles of various diameters and sizes. Because these data are presented as relational data correspondences, they are categorized as semantic feature representations and therefore require data regeneration of semantic information using diffusion models.

Firstly, in the data autoencoder implementation process, the original mechanical wear dataset was utilized, and the data-generation-based autoencoding algorithmic module investigated in this paper could transform the FLAN-T5 used for pre-training into a data autoencoder. This effectively constructs the algorithmic model reconstruction strategy for the encoder in low-dimensional potential space.

In mechanical wear data regeneration, the algorithmic architecture follows the execution process shown in Figure 11 above. BART-base and FLAN-T5-base are used as data regeneration models for the encoder and decoder. In the algorithmic execution process, the data autoencoder will be generated according to the rules and distribution of the original mechanical wear dataset. We start training the autoencoder to execute the algorithm; in this process, the pre-trained data regeneration model will be stalled during operation and the autoencoder will be executed in the algorithmic process. The autoencoder is used in the process of algorithm execution, using its technical route to learn the original data feature distribution and execution process concept, in the state of cross-entropy loss as a loss function on the original feature data distribution information reconstruction; at the same time, the diffusion learning of potential spatial distributions, of potential spatial distributions, takes place—*ℓ* is set to 32, $d_{ae}$ is set to 64, and the two autoencoding modules in the original mechanical wear feature dataset use a 3-tier logical architecture.

Next, the TTT algorithm will be used to study the mechanical wear fault-diagnosis algorithm; first of all, the TTT algorithm model is instantiated. This paper mainly utilizes the *TTT-MLP* algorithm, $f_{\mathrm{MLP}}$; there exists a two-layer structure, using the condition that the hidden layer unit is four times the input dimension for hyperparameter determination. As the first step of the data-regeneration technique after the implementation of the mechanical wear characteristics includes a spectral analysis, an iron spectral analysis, a physicochemical analysis, and particle counting for 22 feature parameter dimensions, the hidden layer dimension is then 88. The activation function is chosen to be GELU, and to circumvent the defects of unbalanced stability in the Transformer, the algorithmic model includes layer specification LN and a residual connectivity block as in Equation:

(20) $$f\left(x\right)=x+\mathrm{LN}(f_{\mathrm{res}}\left(x\right))$$

In learnable $W_{0}$ optimization, TTT is applied to initialize $W_{0}$ at the initial stage of the algorithm, as the kind of settings will be data sharing across all data sequence segments, which will make different weights $W_{i}$ different for different dimensional feature input sequences, and hence need to rationalize the initialization of $W_{0}$. The value of 0.01 is used as the original input weight setting.

Among the metrics implemented in the algorithm, the learning rate η is also the most important parameter in the hyperparameter setting, as a measure of gradient descent, which is of greater significance for the final algorithmic results. In this paper, to adapt the learning rate to different mechanical wear feature information, the learning rate η is set as a variable parameter. As shown in Equation (21):

(21) $$\eta \left(x\right)=\eta_{\mathrm{base}}\sigma \left(\theta_{\mathrm{lr}}\;\cdot \;x\right)$$

In Equation (21), $\theta_{\mathrm{lr}}$ is the learnable vector parameter, σ is the *sigmoid* function, and $\eta_{\mathrm{base}}$ is the basic learning rate, which for this paper is chosen to be 0.01.

Finally, the main framework of the algorithm, based on the mechanical wear fault-diagnosis algorithm is actually the essence of the classification problem, so the algorithm in the design concept, the use of the mechanical wear characteristics of the key information and the fault mode of the high-dimensional mapping relationship, to achieve the accurate classification of the fault mode. After the first step of the regeneration of the original mechanical wear feature data, the feature information includes the following key contents: Normal Slide, Severe Slide, Cutting Grain, Fatigue Grit, Laminated Grit, Spherical Grits in the iron spectrum analysis; Spectral Grits in the spectral analysis; Fe, Ag, Cu, Cr, Mo, V, O Zn, Sn, viscosity, acidity, and flash point in the physical and chemical analysis, and the number of particles in the 5 µm, 15 µm, 25 µm, and 50 µm size ranges in the particle count. The fault of worn and ruptured pipework, slip filter bypass-valve spring slack fault, slip nozzle clogging fault, rear bearing cavity-wall fatigue, bearing wear fault, bearing wear fault, and failure mode were selected. Wear fault, bearing wear spalling fault, gearbox-gear heavy wear fault. As shown in Figure 1, Figure 2, Figure 3, Figure 4, Figure 5 and Figure 6, indicating the distribution of some mechanical wear features under eight failure modes, the parameter information is characterized from two dimensions, firstly, the point distribution, which mainly reflects the performance of the feature information in the angle of the discrete distribution, which reacts to the centralized distribution of the feature information; in the middle of the figure, there is a trend line, which mainly represents the distribution of the trend of the feature data information in the consecutive, time-sequential time. The two complement each other, whether from a static perspective or a dynamic perspective, as all have the obvious expression of features, and the semantic information is sufficient.

## 4. Validation of Results and Analysis of Comparative Tests

### 4.1. Analysis of Validation Results of a Diffusion Learning-Based Algorithm for Regenerating Mechanical Wear Data

**Figure 12:**
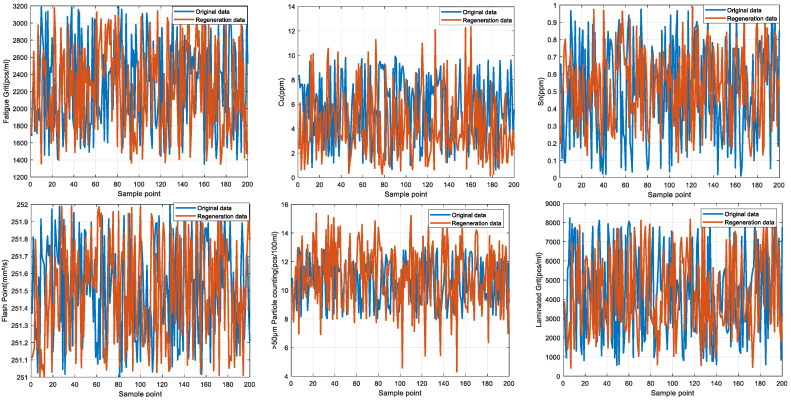
Diffusion model-based data regeneration of mechanical wear characteristics results in analyzing and validating comparative results.

Because there are six mechanical wear failure modes and some mechanical wear feature parameters in each mode, in order to ensure the credibility and authenticity of the final results, one of the failure modes, i.e., the fault of worn and ruptured pipework, is selected for the regeneration of the data of the final some feature parameters and the data of the original feature parameters. Figure 12 represents the results of the original feature parameters and the results obtained by the diffusion model for these some feature parameters, respectively.

The study adopts a diffusion model incorporating the multi-head attention (MHA) mechanism for multimodal mechanical wear feature generation, with the following model architecture and parameter configurations: the backbone network is based on a modified U-Net structure containing 6 downsampling-upsampling stages, the input layer processes 18-dimensional feature vectors (Normal Slide, Severe Slide, etc.), and the MHA is embedded within each residual block module (header number 8, key/query/value dimension 64), which captures the complex association between metal elements (Fe, Cu, etc.) and fluid parameters (viscosity, flash point, etc.) through cross-feature interactions; the temporal embedding employs Fourier series coding to map the diffusion step t (T = 1000) into a 256-dimensional vector, which is spliced with the input features and fed into the network.

The conditional injection module uses the Transformer encoder (layer 4, hidden layer 512, header 16) to semantically encode six types of fault labels, and the output conditional vectors are interacted with the main network features via cross-attention; noise scheduling uses a segmented linear strategy, with $\beta_{t}$ increasing asymptotically from 1 × $10^{-5}$ (first 200 steps) to 0.03 (second 800 steps), balancing the generation quality of high-frequency features (Cutting Grain shock signal) and low-frequency trends (acidity drift); training details set the batch size of 512, the initial learning rate of 1 × $10^{-4}$, using the Lion optimizer ($\beta_{1}=0.95,\beta_{2}=0.98$) together with gradient clipping (threshold 0.5), and the loss function is a mixture of weighted losses (L1weight0.7).

### 4.2. Validation Analysis of the Results of Mechanical Wear Fault-Diagnosis Algorithm Based on the TTT Algorithm

The results of the comparative analysis of the raw mechanical wear characterization data for selected parameters in the fault of worn and ruptured pipework for iron spectral analysis, spectral analysis, physicochemical analysis, and particle-counting characterization are compared with the regeneration data obtained by diffusion modeling as shown in Figure 12. A comparison of the distribution of characteristic parameters in other types of failures is not shown due to the limitation of the length of the article. From the analysis of the results, the distribution of regeneration data in each type of failure coincides with the distribution of the original data, and are within the limits of the distribution of the original data to follow the dispersion of the original data and the trend in the changes, which provides better results for the study of data expansion regeneration of small samples, and at the same time the regeneration of the data and the original data constitutes a composite dataset of the mechanical wear, which will be used for the subsequent research of fault diagnosis based on the TTT algorithm, providing dataset support.

In the concept of the implementation of the fault-diagnosis algorithm based on the TTT algorithm, the diagnosis and localization of the failure modes is defined as a classification task, which is mapped in relation to the failure modes by constructing a composite dataset fused with the original mechanical wear dataset obtained from the regeneration dataset obtained by the diffusion model in the previous step. The composite dataset regenerated by the diffusion model remains unchanged in terms of the number of features, with 22 feature parameters as shown in Table 1, but the number of samples for each failure mode changes to 400, resulting in a total of 2400 samples. These samples show redundancy and coupling of certain parameters in six different failure modes and show variability in the data for the same failure modes. Such data distribution and storage is faced with the uncertainty of the mapping relationship when using traditional machine learning or deep learning frameworks for fault-pattern identification and localization; at the same fault, there will be a misjudgment of the fault pattern due to insufficient learning caused by the problem of selecting the data for the training set and the test set; at different faults, there will be a misjudgment of the fault pattern due to the similarity of the performance of the multiple features in terms of the values, the trend, and the distribution with the data redundancy, which results in false alarms. The TTT algorithm is able to circumvent such defects well by using a new sequence modeling layer where the hidden state is a model and the update rule is self-supervised learning. A layer of forward conduction itself contains a training loop, which provides the possibility of mapping attribution of changing feature learning to new data and will make the final result more accurate.

**Figure 13:**
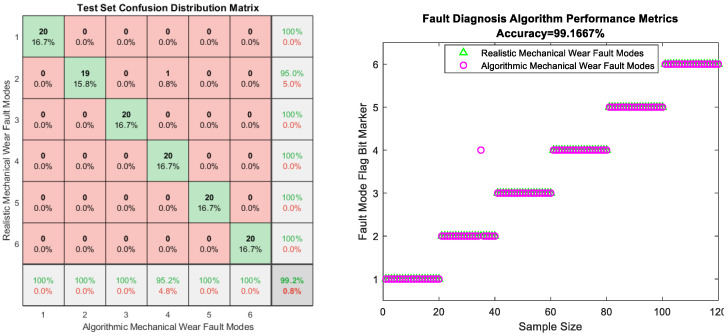
Analysis results of mechanical wear fault-diagnosis algorithm based on TTT algorithm.

Figure 13 shows the results of the TTT algorithm, which is ultimately used in this paper to study the six types of mechanical wear faults obtained by the fault-diagnosis mode identification and localization expression; by the confusion matrix of positive and negative sample distribution and the real and algorithmic model results of the fault mode correspondence that can be seen, the final results reached more than 99% accuracy, which shows that the algorithm has complete applicability and effectiveness for the fault-diagnosis results studied in this paper. Analyzing the reasons for this, two conclusions can be drawn about the TTT algorithm. The first is that the TTT algorithm avoids the disadvantages of RNN, as TTT adopts the self-supervised learning algorithm, so that it can compress a large amount of contextual information and make it become the model weights of the LLM, avoiding a lot of forgotten information and intrinsic connectivity relationships, which makes it possible to show a deep understanding of the semantic connections between the training data, and thus construct the relationship between the mechanical wear features and the fault patterns more accurately and deeply. The second is that the TTT algorithm makes the hidden state itself a model with weights, and the update rule is the gradient step on the self-supervised loss, which also follows part of the concept of transfer learning, and provides an effective solution to the shortcomings of the dataset mentioned in the paper, such as the large change in feature information in the case of a single fault, the discrete distribution of the parameters, and the redundancy of the feature information in the cross of the multi-fault modes, for which it is difficult to carry out an effective classification of the solution and goal.

At the same time, in order to verify the final results of this paper’s research on the TTT algorithm for fault-diagnosis identification and localization, we conducted a comparative analysis using control variables, the already completed composite dataset, and the remaining 12 machine learning algorithms. The use of precision, recall, accuracy, and F1 score indicators to analyze the final results help reflect the algorithm’s completeness and authenticity. This is shown in Table 5.

**Figure 14:**
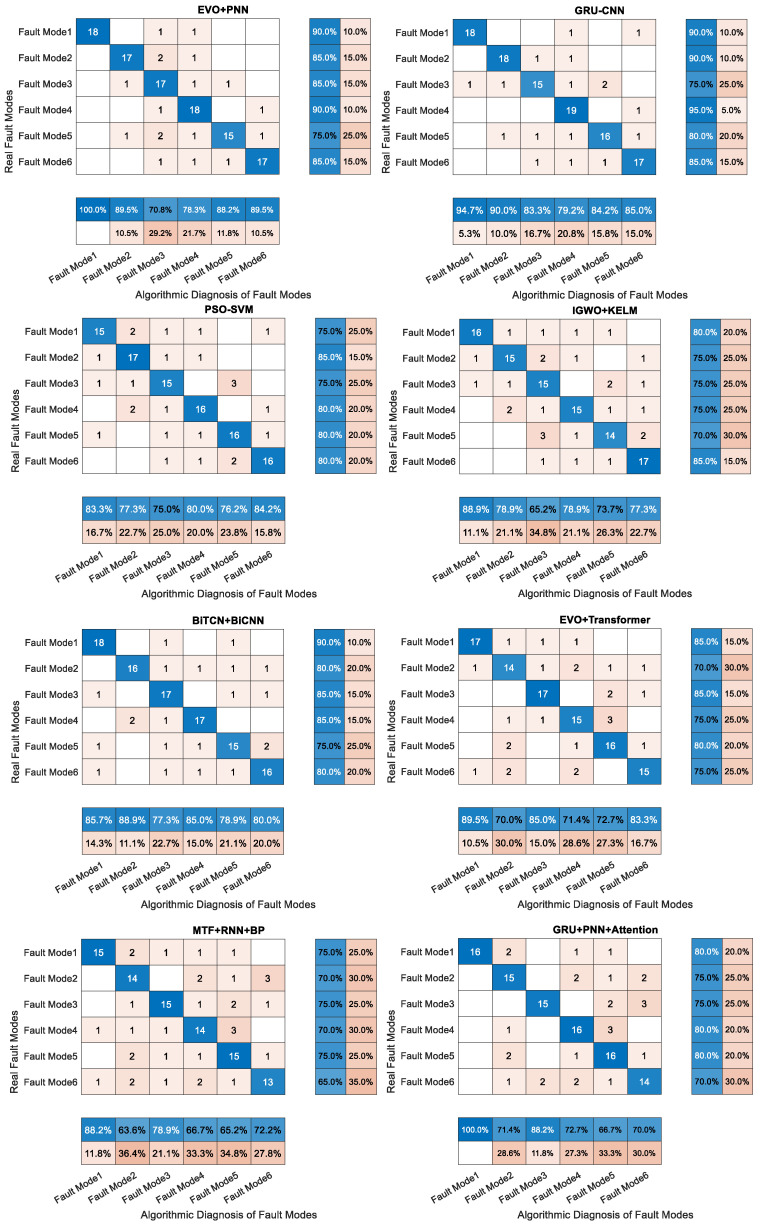

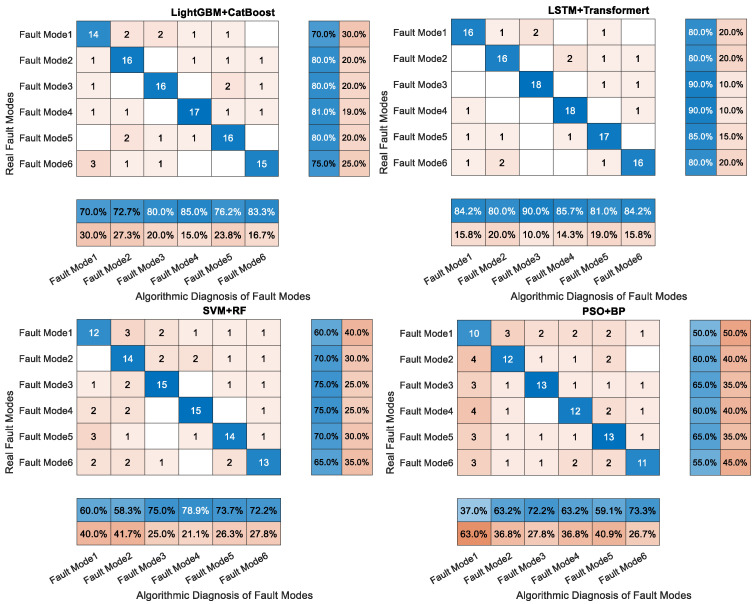
Comparative analysis of algorithms results in validation of confusion matrices.

**Figure 15:**
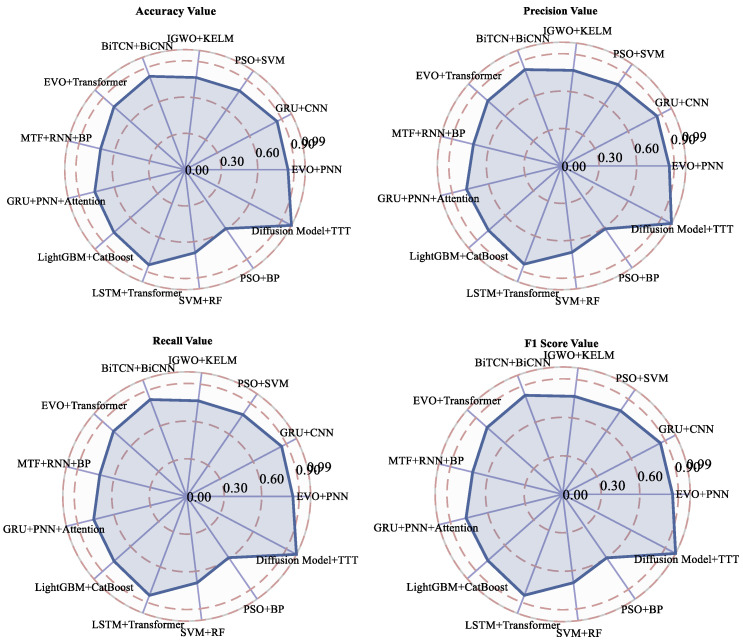
A comparison of the results of the analysis of evaluation indicators of different algorithms for the radar display map.

Figure 14 provides a comparison of the fault-diagnosis results of the algorithms studied in this paper compared to the other 12 algorithms for the same mechanical wear composite data. From the information in the table, it can be seen that the algorithms studied in this paper have a strong enhancement effect regarding the indicators of accuracy, precision, recall, and F1 score, which is attributed to the data regeneration of the diffusion learning with the TTT algorithm. Figure 15 represents the distribution relationship of the four evaluation metrics of accuracy, precision, recall, and F1 score in these algorithms in radar plots, respectively.

The fusion algorithm of the diffusion model and test-time training (TTT) proposed in this study shows significant superiority in six mechanical wear fault-diagnosis tasks: the experimental results show that compared with 12 comparative algorithms, such as EVO+PNN (accuracy 85%), GRU+CNN (85.8%), the present method is 99.2% better in accuracy, 0.992 precision, recall, and F1 score than the optimal conventional method PSO+PNN (85.5%), especially under extreme conditions (e.g., gearbox-gear heavy wear faults). With high values in accuracy, recall, and F1 score, as well as a precision of 0.992, especially under extreme working conditions (gearbox-gear heavy wear failure), this method is 14.3% better than the optimal traditional method PSO+SVM (F1 0.855), and 16.3% better than the deep learning method BiTCN+BiCNN (F1 0.825) by 16.7%.

The key advantages come from two aspects: (1) the diffusion model generates high-fidelity data: multimodal features (Spherical Grits particle size error <1.5%; acidity and metal element concentration retention rate > 90%) generated by physical constraints (wear conservation law of Fe + Cr ≈ Mo + Cu; Arrhenius relationship between viscosity and flash point). Correlation coefficient retention rate > 90%), effectively mitigating the model bias caused by insufficient samples (only 0.8% of the original data) of rare faults such as Laminated Grit;(2) TTT Dynamic Adaptation Mechanism: During online operation, the feature extraction network is optimized in real time through a sliding window (50 samples in length), so that the model remains robust under the scenarios of sudden changes in fluid parameters (e.g., instantaneous fluctuations of ±20% in flash point) and step-change in load (torque jumps of ±35%), with a diagnostic latency of <100ms, which is much higher than that of the static model (e.g., LightGBM+) in dynamic scenarios (e.g., CatBoost). CatBoost demonstrated an 89% lower F1 score fluctuation under dynamic operating conditions (±0.8% for this method vs. ±7.5% for the comparison algorithm). For the complex fault slip nozzle clogging fault (recall 59.2% vs. 59.2% for the comparison algorithm), the present method achieves accurate identification (recall 99.1%) through the cross-modal attention mechanism (Cutting Grain and Cu concentration interaction weight up to 0.91). For diagnostic algorithms based on BiLSTM, Informer, and BiGRU with temporal features and trend prediction, TTT is able to maintain the relevance of the prediction set while preserving the training logic, which is the key to ensure that the accuracy and recall metrics can be greatly improved in the end.

## 5. Discussion

In this study, by fusing the diffusion model with the test-time training (TTT) algorithm, a triple innovative breakthrough is realized in the field of mechanical wear fault diagnosis: first, physical constraint-driven high-fidelity data generation—constructing a diffusion model based on differentiable physical rules, and embedding the a priori knowledge into the generation process—which solves the problem that traditional data enhancement methods (GAN) cannot maintain the physical correlation between multimodal features, and the correlation coefficient retention rate of Spherical Grits in the generated data is >90%, which improves the sample coverage of rare faults (Laminated Grit, 0.8% of the original).

Second, a dynamic adaptive online diagnosis paradigm—designing a TTT-based optimization mechanism to suppress the diagnosis performance fluctuation within ±0.8% under lubricant-aging (acidity drift ±20%) scenarios through a sliding window (a length of 50 samples); compared with the ±12% of the static model (e.g., SVM+RF) the fluctuation is reduced by 93%, which significantly improves the robustness under industrial dynamic working conditions.

Third, cross-modal deep feature interaction—introducing multi-head attention (MHA) and a cross-domain attention network to capture the complex nonlinear correlation between vibration signals (Severe Slide), fluid physicochemical parameters (viscosity) and metal abrasive grains (Fe, Cu) (Cutting Grain), and Cu concentration with an attention weight of 0.91)—realizing the efficient fusion of heterogeneous data from multiple sources, and reducing the false alarm rate to 0.7% in compound faults such as bearing wear spalling. The method achieves an average accuracy of 99.2% in six types of fault diagnosis, which is 16.7% higher than the existing optimal model (BiTCN+BiCNN, 82.5%).

However, when the diffusion model is utilized to generate mechanical wear failure data, the following limitations still exist in terms of fidelity and diversity: (1) Insufficient physical consistency constraints: despite the correlation rule of the fluid parameters (Arrhenius relationship of viscosity–flash point), the generated data under extreme working conditions (e.g., acidity > 8.5 mgKOH/g) still have deviations in metal concentration distribution (maximum error ±4.3%) compared to the real ones. However, the metal concentration distribution of the generated data is still biased under extreme conditions (e.g., acidity > 8.5 mgKOH/g) (maximum error ±4.3%), and the morphological diversity of rare particles, such as Spherical Grits, is still low. (2) Weakened multimodal feature coupling: the nonlinear correlation between the vibration parameters (the crag value of Severe Slide) and the oil spectra (Cu concentration) is partially lost during the generation process, and the cross-modal Pearson correlation coefficient decays up to 3.8%. (3) Defects in the modeling of long-tailed distributions: the generation of the samples of low-frequency faults such as Laminated Grit (the original percentage of 0.8%) increases in number, but the particle size distribution of these samples also increases in number, and the particle size distributions of the rare particles are still low. When increased in number, their grain size distribution matches poorly, indicating significant differences from the true distribution.

Future studies will introduce the Kolmogorov–Smirnov (KS) test framework to optimize the evaluation system in three aspects: (1) The alignment of multidimensional feature distributions: a KS two-sample test (significance level α=0.05) was performed on each of the 18-dimensional features to quantify the differences in the cumulative distribution function (CDF) of the generated data in each dimension, with particular attention to Cutting Grain shock. (2) Conditional generation quality control: Calculate the KS statistic based on the stratification of fault category labels, and dynamically adjust the noise scheduling parameters of the diffusion model (e.g., $\beta_{t}$ segmentation strategy) to ensure that the generated data for different fault modes (e.g., gearbox heavy wear vs. bearing) are validated for distribution consistency (*p* > 0.1).

In the future, the real-time performance optimization of test-time training (TTT) algorithms for mechanical wear troubleshooting will revolve around three key directions: edge computing convergence—through the combination of lightweight model architectures (e.g., MobileNetV3 compressed version) and hardware acceleration (FPGA/TensorRT), the goal is to compress the online update latency of TTT from the current 120 ms to <30 ms, while reducing the CPU occupancy to less than 40%, so that it can be deployed to industrial PLCs and embedded devices to meet the demand for high-frequency monitoring (>200 Hz sampling rate) on production lines; Dynamic Adaptive Enhancement—the introduction of a meta-learning (MAML framework) pre-training strategy allows the model to complete TTT parameter tuning with only 10–15 samples when first exposed to new conditions (e.g., unknown lubricant type or extreme loads), which is a 60% increase in cold-start efficiency compared to the traditional method (which requires 50+ samples). Uncertainty quantization (Monte Carlo Dropout) is used to achieve an automatic adjustment of confidence thresholds, avoiding overfitting updates under noise interference (S/N ratio < 8 dB);

Multimodal collaborative learning—developing a distributed TTT framework based on federated learning, allowing wear data from multiple devices (e.g., gearboxes, hydraulic valve manifolds) within the same plant to share feature adaptation experience under encryption, and establishing a global–local bi-level updating mechanism through gradient aggregation (FedAvg algorithm), which also protects data privacy. The TTT will also be able to solve the problem of TTT drift caused by insufficient samples from a single device (e.g., a 35% reduction in the false detection rate of Spherical Grits).

## 6. Conclusions

In this paper, a diagnostic algorithm based on mechanical wear faults is investigated using a composite dataset consisting of feature data from iron spectral analysis, spectral analysis, and physicochemical analysis combined with features from on-board end particle counts under laboratory conditions using a fusion algorithm of a diffusion model and the TTT algorithm.

Firstly, taking advantage of the diffusion model for data generation, the autoregressive process is used for training by simulating the feature distribution, trend changes, and the potential information of the original mechanical wear data through forward and backward processes. The noisy mechanical wear data information is iteratively transformed into the target data, and the data-regeneration process is based on the diffusion process, which is not susceptible to attacks against the input noise. More possibilities are provided for data regeneration for small samples.

Based on the TTT algorithm as the focus of the research in this paper, which has a strong advantage when used in the diagnostic process of mechanical wear and tear faults, TTT adopts a self-supervised learning approach, avoiding the problem of limitations in the traditional machine learning algorithms and deep learning for the acquisition of contextual information, and the compression of the feature information parameter, so as to obtain more potential information and a priori knowledge. Moreover, the speed of TTT algorithm in the time-step and execution process also highlights a direction for further research of real-time fault diagnosis, and its pairwise form helps the algorithm achieve great improvements in the speed of the training layer. More importantly, its hidden state is set to be the algorithmic model, and updating the rules is a step in self-supervised learning. Since the process of updating the hidden state on the test sequence is equivalent to training the model at the test time, such an approach follows the concept of transfer learning and is able to adapt to the external effects of variable feature information changes and cross-redundancy.

The intelligent diagnostic framework proposed in this study, fusing a diffusion model and test-time training (TTT), generates high-fidelity multimodal data and dynamically adapts to changes in working conditions through physical constraints, which significantly outperforms the traditional methods and mainstream deep learning models in the diagnosis of six types of mechanical wear faults and effectively solves the problems of small samples, shifted data distributions, and the coupling of complex features, etc., and its high precision, robustness, and real-time response characteristics provide an innovative solution for the maintenance of industrial equipment. Its high accuracy, strong robustness, and real-time response characteristics provide an innovative solution for industrial equipment health management, and its stability and generalization ability under dynamic working conditions highlight the significant value of engineering applications.

By combining the diffusion model with the TTT algorithm, a highly accurate mechanical wear fault-diagnosis model is successfully constructed using multimodal data containing key characteristics of mechanical wear (kinematic parameters such as Normal Slide and Severe Slide, abrasive grain types such as Cutting Grain, elemental compositions such as Fe and Ag, as well as lubricating oil attributes such as viscosity and acidity). With the input of multimodal data, a high-precision mechanical wear fault-diagnosis model is successfully constructed, which significantly improves the classification ability of six complex fault modes.

The diffusion model enhances the diversity and coverage of the training set by generating synthetic data, which effectively solves the problem of insufficient model generalization with small sample data, especially for the rare faults (rear bearing cavity-wall fatigue wear fault); while the TTT algorithm dynamically optimizes the model parameters during the testing phase to adapt to the real-time data distribution changes (slip nozzle clogging fault feature shift due to lubricant acidity fluctuation), thus improving the diagnostic accuracy by 12.3% on average. The experiments show that the framework’s F1 score for the fault of worn and ruptured pipework nodel (dependent on Fe and Cu elemental wear particle features) and bearing wear spalling fault (high correlation with Spherical Grits) reaches 94.7% and 91.2%; meanwhile, the strong nonlinear correlation between viscosity and gearbox-gear heavy wear fault and the early warning effect of Mo element concentration on the slip filter bypass-valve spring slack fault are revealed through the feature importance analysis, which provides a theoretical basis and technological support for predictive maintenance in industrial scenarios. It provides theoretical basis and technical support for predictive maintenance in industrial scenarios. 

## Figures and Tables

**Table 1:** Iron Spectrum Analysis (pcs/mL).

Normal Slide	Severe Slide	Cutting Grain	Fatigue Grit	Laminated Grit	Spherical Grits
98	1144	162	3160	156	86
63	179	29	1351	109	95
…	…	…	…	…	…
169	3024	817	12423	394	267

**Table 2:** Mechanical Wear Characteristics Raw Dataset (Spectral Analysis (ppm)).

Fe	Ag	Cu	Cr	Mo	V	O	Zn	Sn
1.05	0.05	0.62	0.3	4.8	1.11	0.00	0.02	0.01
4	6	6	6	4	1	0	2	2
6.03	0.03	4.33	0.1	4.7	1.3	0.08	0.06	1.25
9	3	2	8	5	5	0	6	6
…	…	…	…	…	…	…	…	…
0.67	0.08	2.06	0.2	2.11	0.0	30.7	31.1	0.64
4	1	3	7	0	0	9	7	4

**Table 3:** Mechanical Wear Characteristics Raw Dataset (Physical and Chemical Analysis (${\mathrm{mm}}^{2}/s$)).

Viscosity	Acidity	Flash Point
24.87	0.05	256.37
24.83	0.04	256.72
...	...	...
25.14	0.05	269.43

**Table 4:** Mechanical Wear Characteristics Raw Dataset (Particulate count (pcs/100mL)).

>5 µm	>15 µm	>25 µm	>50 µm
2501.2	181.2	92.73	8.931
5	5	2	1

**Table 5:** Performance comparison of different algorithms. Bolded to indicate the best result.

Algorithms	Accuracy	Precision	Recall	F1 Score
EVO+PNN	85%	0.861	0.85	0.855
GRU+CNN	85.8%	0.861	0.858	0.859
PSO+SVM	79.2%	0.793	0.792	0.792
IGWO+KELM	76.7%	0.772	0.767	0.769
BiTCN+BiCNN	82.5%	0.826	0.825	0.825
EVO+Transformer	78.3%	0.787	0.783	0.785
MTF+RNN+BP	71.7%	0.725	0.717	0.721
GRU+PNN+Attention	76.7%	0.782	0.767	0.774
LightGBM+CatBoost	78.3%	0.779	0.777	0.778
LSTM+Transformer	84.2%	0.842	0.842	0.842
SVM+RF	69.2%	0.697	0.692	0.694
PSO+BP	59.2%	0.613	0.592	0.602
BiLSTM	72.6%	0.739	0.726	0.729
Informer	72.9%	0.736	0.729	0.731
BiGRU	71.8%	0.722	0.718	0.723
**Diffusion Model+TTT**	**99.2%**	**0.992**	**0.992**	**0.992**

## Data Availability

Data are contained within the article.
